# Granulomatosis with polyangiitis-associated choroiditis simulating serpiginous choroiditis

**DOI:** 10.1016/j.ajoc.2026.102673

**Published:** 2026-09-25

**Authors:** Matthew Lin, Ariel Schlaen, Ramana Moorthy, Toby Steele, Joseph N. Mehrabi, Kareem Moussa

**Affiliations:** a UC Davis Eye Center, University of California Davis, Sacramento, CA, USA; b Department of Ophthalmology, Hospital de Clinicas Jose de San Martin, Universidad de Buenos Aires, Buenos Aires, Argentina; c Associated Vitreoretinal and Uveitis Consultants, Carmel, IN, USA; d Department of Ophthalmology, Indiana University, Indianapolis, IN, USA; e Department of Otolaryngology, Head and Neck Surgery, University of California Davis, Sacramento, CA, USA; f Department of Rheumatology, University of California Davis, Sacramento, CA, USA

**Keywords:** Serpiginous-like choroiditis, Granulomatosis with polyangiitis, Seronegative vasculitis, Choroiditis, Multimodal imaging

## Abstract

**Purpose:**

To describe a rare presentation of bilateral choroiditis simulating serpiginous choroiditis as an initial ocular manifestation of granulomatosis with polyangiitis (GPA) and highlight clinical features that should prompt consideration of this diagnosis.

**Observations:**

A 73-year-old female with renal failure of unknown etiology presented with bilateral vision loss. Fundus examination revealed peripapillary creamy yellow-white choroidal lesions with adjacent atrophy in both eyes. Multimodal imaging demonstrated bilateral choroidal thickening and outer retinal and retinal pigment epithelium atrophy that spared the fovea in the left eye. Review of systems was notable for constitutional, upper and lower respiratory, and otologic symptoms. Extensive infectious and autoimmune testing, including antineutrophil cytoplasmic antibodies, was initially negative. Despite initially negative serologic and biopsy findings, the patient later developed a saddle nose deformity and pulmonary nodules, leading to a diagnosis of seronegative GPA with renal, pulmonary, sinus, and ocular involvement. Treatment with high-dose systemic corticosteroids and local intravitreal corticosteroid implants resulted in quiescence of choroiditis and stabilization of visual acuity over 14 months of follow-up. During a subsequent hospitalization, she demonstrated seroconversion with positive myeloperoxidase (MPO) antibodies, confirming seropositive GPA.

**Conclusions:**

Choroiditis is an uncommon posterior segment manifestation of GPA that may mimic infectious or idiopathic inflammatory chorioretinal diseases. This case underscores the importance of considering GPA in patients with choroiditis and systemic features of vasculitis, even in the absence of initial serologic confirmation, as early recognition and treatment are critical to preserving vision.

## 1 Introduction

Granulomatosis with polyangiitis (GPA), formerly known as Wegener's granulomatosis, is a rare autoimmune disorder characterized by granulomatous inflammation, tissue necrosis, and vasculitis of small-to-medium-sized blood vessels. This systemic condition commonly affects the upper respiratory tract, lower respiratory tract, and kidneys, presenting with sinusitis, pulmonary lesions, renal impairment, fever, and fatigue, among other symptoms.^1^ In 30-50% of patients, GPA also has ocular manifestations, such as conjunctivitis, keratitis, episcleritis, scleritis, and orbital inflammation.^2^ Less commonly, it affects the retina and choroid, leading to vasculitis-associated choroidal infarcts, retinal vascular occlusions, and serous retinal detachment.^3^ Choroiditis simulating serpiginous choroiditis is a much lesser known manifestation of ocular involvement in GPA.^3^^,^^4^ In this report, we present a case of bilateral choroiditis simulating serpiginous choroiditis in a patient with GPA and discuss the relevant features that should raise suspicion for GPA as the etiology.

## 2 Case

A 73-year-old female with renal failure of unknown etiology and bilateral vision loss was referred for evaluation. Visual acuity was hand motion in the right eye and 20/40 in the left eye. Intraocular pressure was normal in both eyes, and no afferent pupillary defect was noted. Anterior segment exam was unremarkable. Fundus examination was notable for cells in the anterior vitreous, peripapillary creamy yellow-white choroidal lesions with adjacent patches of atrophy in the posterior pole in both eyes, and a macular hemorrhage in the left eye (Fig. 1A–D). Optical coherence tomography (OCT) revealed choroidal thickening in both eyes, and outer retinal and retinal pigment epithelium (RPE) atrophy involving the right fovea and sparing the left fovea (Fig. 1E and F). Fluorescein angiography was notable for mild peripapillary leakage and surrounding staining in the posterior pole in both eyes (Fig. 1G and H). Indocyanine green angiography revealed diffuse hypofluorescence in the posterior pole corresponding to the areas of outer retinal and RPE atrophy and choroiditis on examination in both eyes (Fig. 1I and J).

**Fig. 1:**
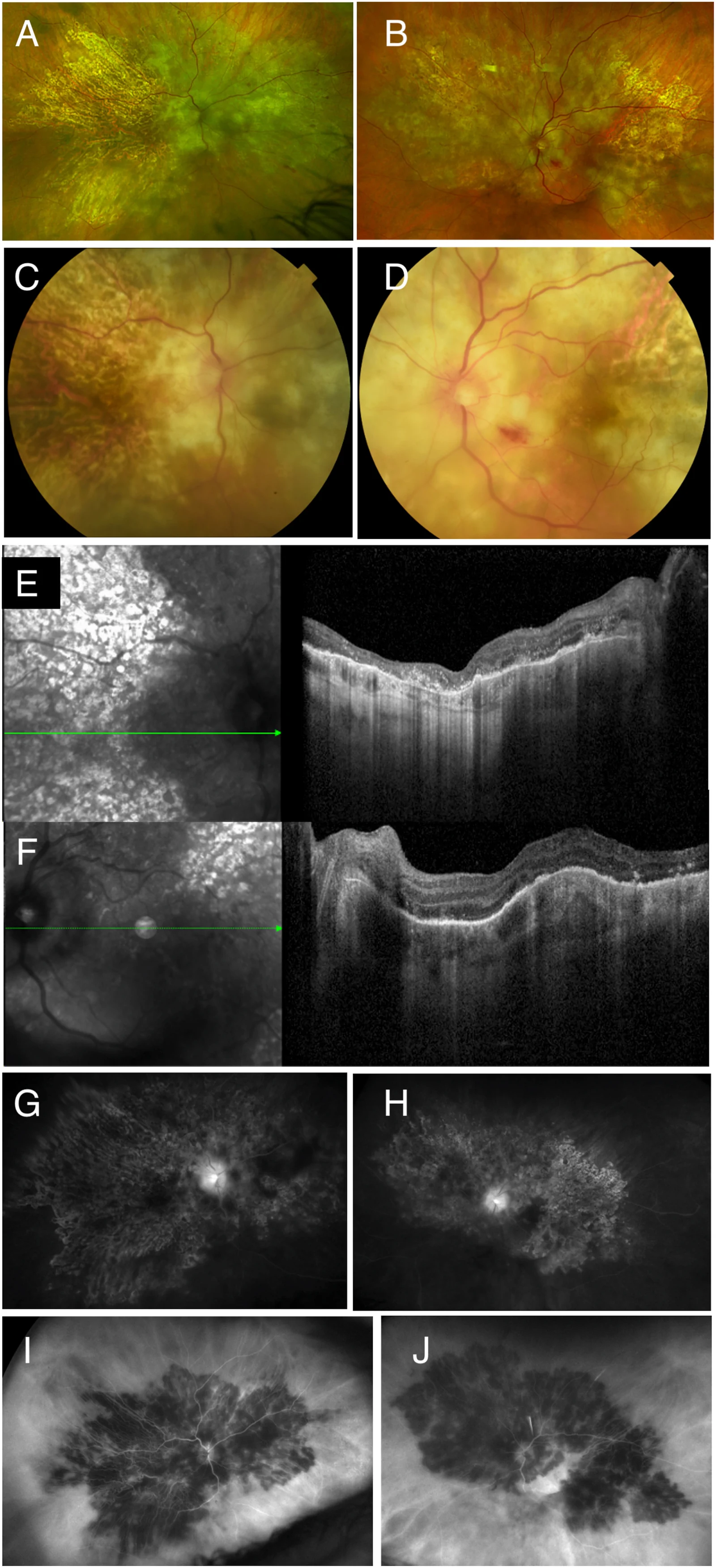
Imaging at presentation. (A-D) Color fundus photography shows peripapillary creamy yellow-white choroidal lesions with adjacent patches of atrophy OU and macular hemorrhage OS; (E, F) OCT-macula shows choroidal thickening OU, and extensive outer retinal and RPE atrophy involving the fovea OD and sparing the fovea OS; (G, H) Fluorescein angiography shows mild peripapillary leakage and surrounding staining in the posterior pole OU; (I, J) Indocyanine green angiography shows diffuse hypofluorescence of the posterior pole in the region of outer retinal and RPE atrophy and choroiditis OU.

On further investigation, the patient reported weight loss, jaw fatigue, productive cough with intermittent blood, joint pain with accompanying early morning stiffness, recurrent epistaxis, chronic sinusitis, and chronic left ear hearing loss. The patient was admitted to the hospital for expedited work-up. A temporal artery biopsy was negative for giant cell arteritis. Serum rheumatoid factor, anti-neurophil cytoplasmic antibodies (ANCA), syphilis antibody, rapid plasma regain, and interferon gamma release assay were negative or within the normal reference range. Computed tomography of the chest revealed bilateral peri-broncho-vascular ground-glass opacities and scattered nonspecific calcified granulomas. Magnetic resonance imaging of the face revealed severe mucosal thickening of ethmoid air cells, sphenoid sinuses, and maxillary sinuses, and sinus biopsy revealed nonspecific chronic and acute inflammation considered negative for GPA. She was treated with a 3-day course of intravenous methylprednisolone (1 g daily) followed by an oral prednisone taper over several months, as well as intravitreal dexamethasone implant (0.7mg) injections in both eyes. Several months later, she developed a saddle nose deformity as well as pulmonary nodules, ultimately leading to a diagnosis of seronegative granulomatosis with polyangiitis with involvement of both the upper (sinusitis) and lower (pulmonary nodules) respiratory tract, kidneys, and eyes (choroiditis) (Fig. 2A and B).

**Fig. 2:**
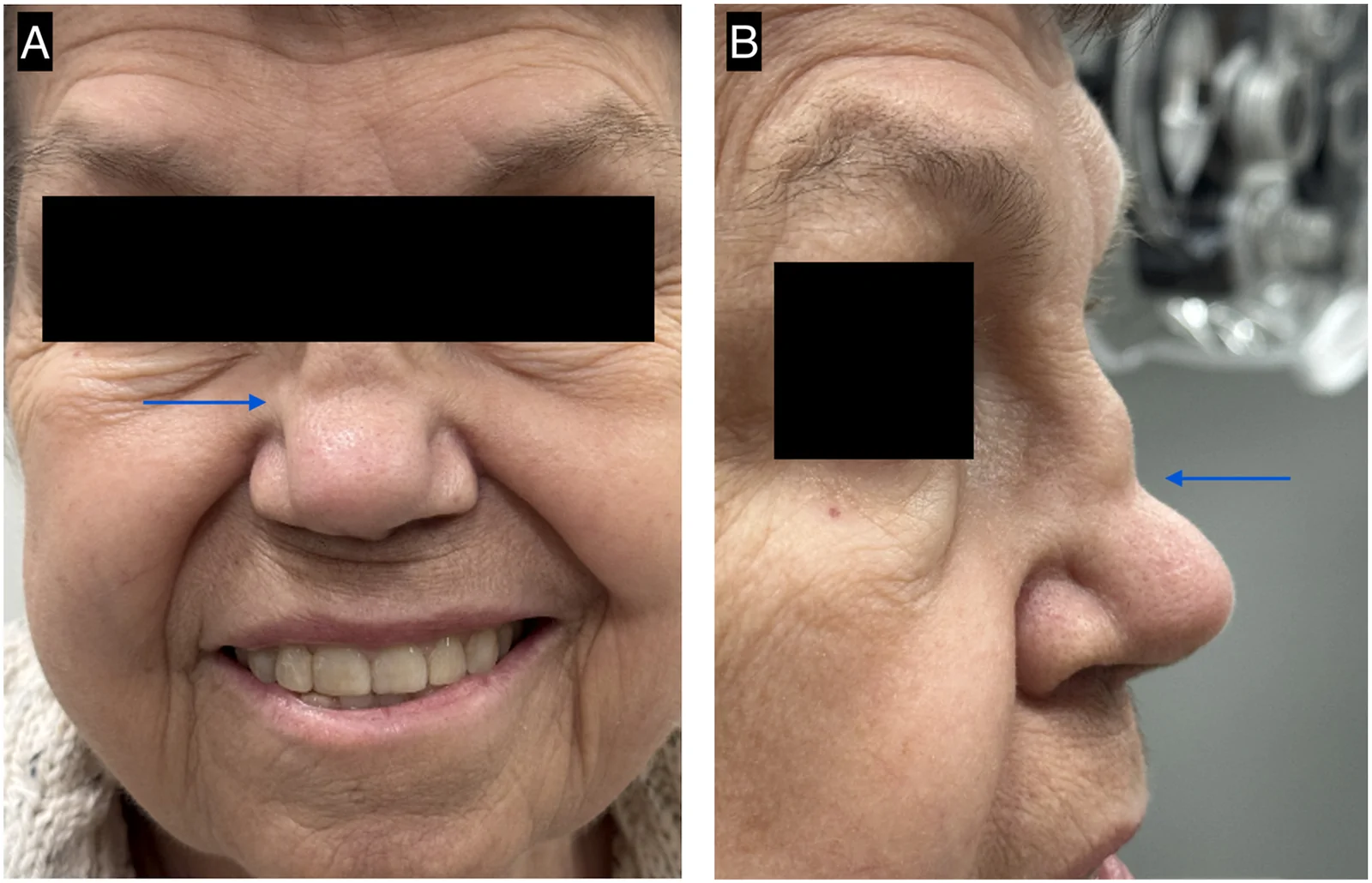
Clinical photographs taken 9 months after initial presentation to ophthalmology. (A, B) A saddle nose deformity (arrows), not present at prior visits, is now evident on anterior and lateral views of the face.

At last follow-up 14 months after initial presentation, visual acuity was stable at hand motion in the right eye and 20/25 in the left eye, with normal intraocular pressures in both eyes. She had received treatment with a total of two intravitreal dexamethasone implants (0.7mg) in each eye followed by intravitreal fluocinolone acetonide (0.19mg) implant in each eye. The choroiditis in both eyes was inactive, without further expansion of the atrophic patches noted at presentation (Fig. 3A–D).

**Fig. 3:**
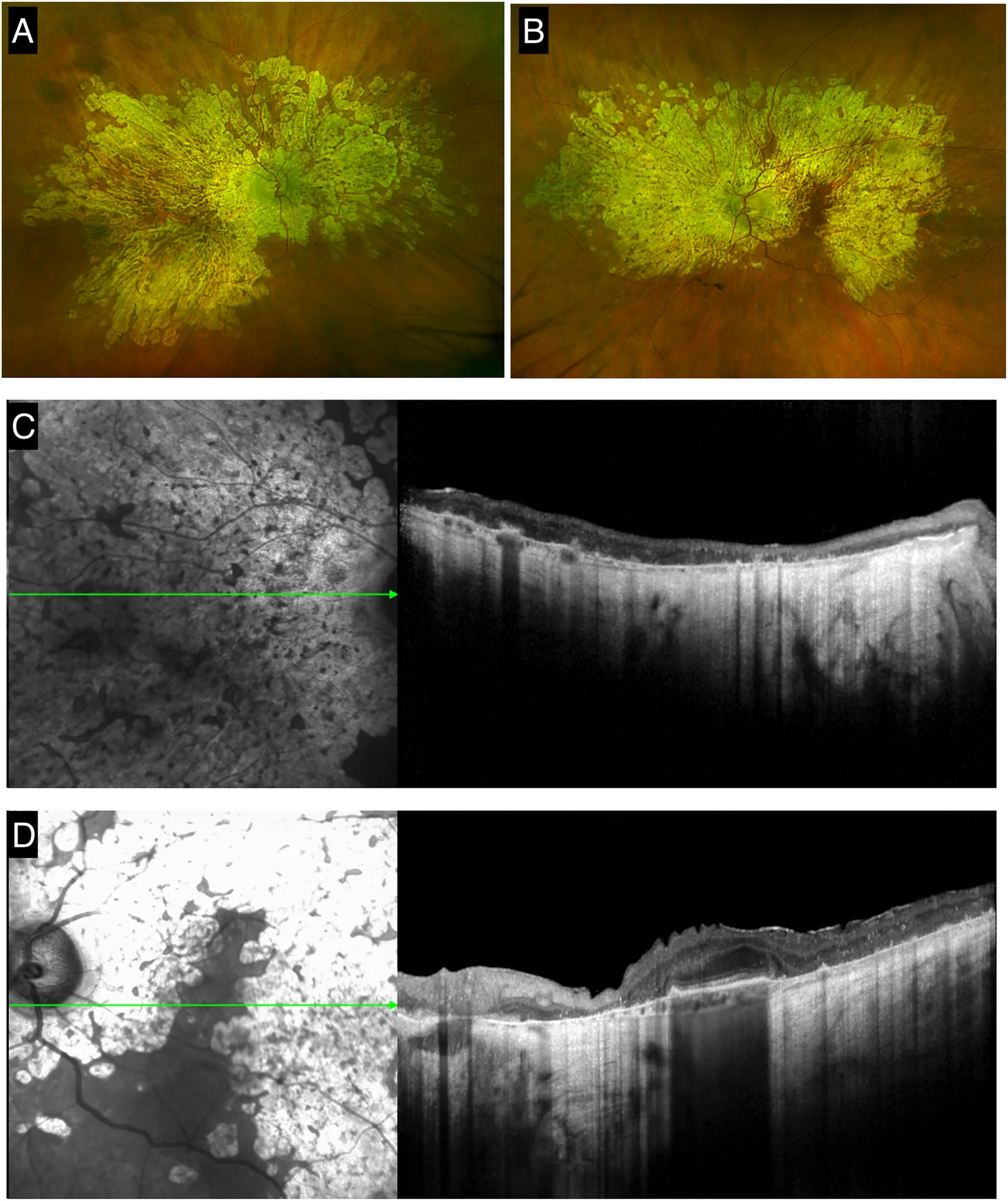
Repeat imaging 14 months after initial presentation, having received systemic steroids, two rounds of intravitreal dexamethasone implants OU, and intravitreal fluocinolone acetonide implant OU. (A-B) Color fundus photography shows stable chorioretinal atrophy centered around the nerve, involving the entire macula OD and sparing the fovea OS; (C-D) OCT-macula shows resolution of choroidal thickening and diffuse outer retinal and RPE atrophy, sparing the central fovea OS.

Following her last ophthalmic follow-up, the patient was hospitalized for worsening systemic disease and initiation of rituximab therapy for presumed seronegative granulomatosis with polyangiitis. During this admission, repeat serologic testing demonstrated seroconversion with positive myeloperoxidase (MPO) antibodies. Her hospital course was subsequently complicated by bacterial peritonitis related to peritoneal dialysis, and she unfortunately passed away.

## 3 Discussion

GPA is a systemic necrotizing vasculitis that most commonly involves the upper and lower respiratory tract and kidneys and may affect the eye in up to 30–50% of patients.^1^ Ocular manifestations frequently involve the anterior segment and adnexa, with scleritis and episcleritis being among the most common and characteristic findings. Posterior segment involvement is less frequent and includes retinal vasculitis, choroidal infarction, serous retinal detachment, and, more rarely, choroiditis. Because posterior segment manifestations of GPA are uncommon and can mimic other inflammatory chorioretinal diseases, recognition of this entity requires a high index of suspicion, particularly in patients with systemic symptoms suggestive of vasculitis.

Serpiginous choroiditis is a rare, chronic inflammatory disease characterized by geographic, serpentine lesions that typically originate in the peripapillary region and extend centrifugally, with relative absence of vitreous inflammation and a tendency for recurrent activity at lesion borders.^5^ Serpiginous-like, or serpiginoid, choroiditis represents a distinct clinical entity that shares a similar fundus appearance but differs in several important respects. Serpiginous-like choroiditis is often multifocal, may exhibit more prominent vitreous inflammation, and features choroidal thickening on enhanced-depth imaging OCT.^6^^,^^7^ Importantly, serpiginous-like choroiditis is classically linked to infectious etiologies, most notably *Mycobacterium tuberculosis*, and distinguishing between these entities is critical, as management differs substantially. Whereas idiopathic serpiginous choroiditis is typically treated with immunosuppression, serpiginous-like choroiditis associated with tuberculosis requires anti-tuberculous therapy, often in conjunction with corticosteroids.^6^^,^^7^ In the present case, the hypofluorescence noted on ICG angiography and the overlying outer retinal/RPE loss likely represents GPA-associated vasculitic choroidal infarction with secondary geographic atrophy. While tuberculosis remains a key consideration in patients presenting with serpiginous-like choroiditis, this case, along with previously reported cases, highlights that GPA should also be considered in the differential of cases resembling serpiginous choroiditis, particularly when ocular findings are accompanied by respiratory, renal, or systemic inflammatory symptoms.

Posterior segment findings in GPA may also occur secondary to posterior scleritis, which can lead to choroidal thickening, subretinal fluid, and serous retinal detachment, as demonstrated by a separate case illustrated in Fig. 4A–C.^8^ However, the mechanism in such cases is distinct, with choroidal and retinal changes occurring secondary to scleral inflammation rather than primary choroidal disease. In the present case, there were no clinical or imaging findings suggestive of scleritis, supporting a diagnosis of primary choroiditis rather than posterior scleritis–related changes. This distinction is important, as it highlights the heterogeneity of posterior segment involvement in GPA and the need for careful clinical and imaging evaluation.

**Fig. 4:**
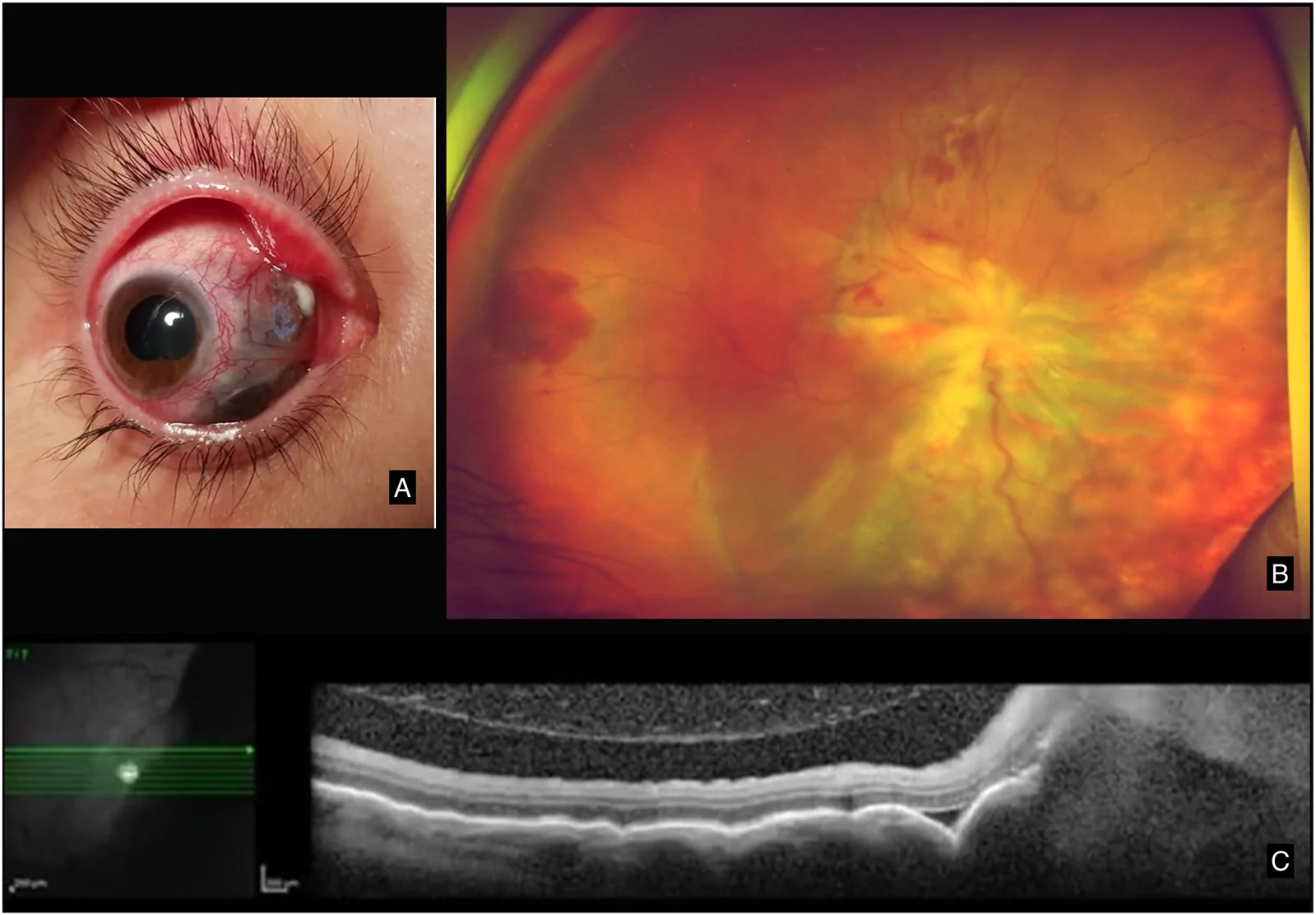
33-year-old female with GPA and necrotizing scleritis of the right eye involving both the anterior and posterior sclera, with secondary choroidal thickening and exudative retinal detachment. (A) Necrotizing scleritis (arrow); (B) Color fundus photography shows exudative retinal detachment; (C) OCT demonstrates choroidal thickening.

Finally, this case highlights the importance of expedited systemic work-up and prompt initiation of therapy in patients presenting with inflammatory choroiditis, particularly when the fovea is threatened. Early recognition of an underlying systemic vasculitis allowed for timely initiation of high-dose systemic corticosteroids, as well as local corticosteroid therapy, with stabilization of visual acuity and prevention of further lesion expansion. While infectious etiologies such as tuberculosis should be considered as mimickers of serpiginous choroiditis, GPA should also be considered in the differential diagnosis, particularly when ocular findings are accompanied by systemic features suggestive of vasculitis, including respiratory and renal involvement.

## CRediT authorship contribution statement

**Matthew Lin:** Writing – review & editing, Writing – original draft, Visualization, Investigation, Conceptualization. **Ariel Schlaen:** Writing – review & editing, Visualization, Supervision, Conceptualization. **Ramana Moorthy:** Writing – review & editing, Supervision, Conceptualization. **Toby Steele:** Writing – review & editing, Investigation, Conceptualization. **Joseph N. Mehrabi:** Writing – review & editing, Investigation, Conceptualization. **Kareem Moussa:** Writing – review & editing, Writing – original draft, Visualization, Supervision, Investigation, Conceptualization.

## Patient consent

Written consent to publish these case details was obtained from the patient.

## Authorship

All authors attest that they meet the current ICMJE criteria for Authorship.

## Funding

No funding or grant support was received for this manuscript.

## Declaration of competing interest

The authors declare that they have no known competing financial interests or personal relationships that could have appeared to influence the work reported in this paper.
